# Cataract Surgery in Eyes with Congenital Iris–Choroidal Coloboma: Quantitative Assessment of Intraocular Lens Alignment and Optical Quality

**DOI:** 10.3390/jcm15176616

**Published:** 2026-08-27

**Authors:** Yuxuan Jia, Enjie Li, Zhenyu Wang, Xijin Wu, Xuefei Ding, Qi Zhang, Ting Chen, Peilin Yue, Xudong Song

**Affiliations:** Beijing Tongren Eye Center, Beijing Tongren Hospital, Beijing Ophthalmology and Visual Science Key Laboratory, Capital Medical University, Beijing 100730, China; jyx18803129393@163.com (Y.J.); lienjie0726@163.com (E.L.); wzy19940825@163.com (Z.W.); xijinwu06@163.com (X.W.); dingxfxf@icloud.com (X.D.); 15194270175@163.com (Q.Z.); tingchen0829@163.com (T.C.); yuepeilin@126.com (P.Y.)

**Keywords:** cataract surgery, congenital iris–choroidal coloboma, intraocular lens alignment, optical quality, phacoemulsification

## Abstract

**Background/Objectives:** Cataract surgery in eyes with congenital iris–choroidal coloboma is technically challenging, and postoperative intraocular lens (IOL) alignment and objective optical quality remain insufficiently characterized. This study evaluated short-term surgical outcomes, early postoperative IOL alignment, and objective optical quality after phacoemulsification with IOL implantation in eyes with congenital iris–choroidal coloboma and cataract. **Methods:** This retrospective comparative study included 36 eyes with congenital iris–choroidal coloboma complicated by cataract and 31 eyes with age-related cataract. All eyes underwent phacoemulsification with in-the-bag monofocal IOL implantation. Best-corrected visual acuity (BCVA), intraocular pressure (IOP), early postoperative IOL tilt and decentration, and objective optical quality parameters, including modulation transfer function (MTF) and Strehl ratio (SR), were evaluated 1 month postoperatively. Multivariable linear regression analyses were performed to identify factors associated with postoperative BCVA and IOL tilt in coloboma eyes. **Results:** BCVA improved significantly after surgery in coloboma eyes (*p* < 0.001), although postoperative BCVA remained worse than that in age-related cataract eyes after adjustment for age (*p* < 0.001). IOP showed no statistically significant change in either group. Coloboma eyes exhibited greater IOL tilt and decentration than age-related cataract eyes (7.60 vs. 4.10° and 0.40 vs. 0.14 mm, respectively; age-adjusted *p* < 0.001 and *p* = 0.004), with lower MTF and SR (among eyes with evaluable aberrometry, 17 coloboma eyes and 30 age-related cataract eyes, all *p* < 0.001). Macular and/or optic disc involvement was associated with worse postoperative BCVA and greater postoperative IOL tilt. **Conclusions:** Phacoemulsification with IOL implantation was associated with short-term visual acuity improvement in eyes with congenital iris–choroidal coloboma. These eyes showed greater early postoperative IOL tilt and decentration than age-related cataract eyes, while the evaluable subgroup showed poorer objective optical quality. Macular and/or optic-disc involvement was associated with worse postoperative BCVA and greater IOL tilt.

## 1. Introduction

Congenital iris–choroidal coloboma is a developmental ocular anomaly resulting from incomplete closure of the embryonic fissure [1]. It may occur as an isolated ocular condition or in association with systemic developmental abnormalities. The reported prevalence ranges from approximately 2 to 14 cases per 100,000 individuals, with most cases occurring sporadically [2]. This congenital malformation is frequently associated with cataract, glaucoma, microphthalmia, nystagmus, amblyopia, refractive errors, and optic nerve atrophy [3,4,5,6]. Among these associated conditions, cataract is the most common, affecting approximately 16% to 50% of patients [6].

Cataract surgery remains the only effective treatment for patients with congenital iris–choroidal coloboma coexisting with cataract and is most commonly performed using phacoemulsification with intraocular lens (IOL) implantation [7,8,9,10]. However, surgical management in these eyes is technically demanding due to distinctive anatomical abnormalities, such as iris defects, zonular weakness, and abnormal capsular support. These structural alterations substantially increase surgical complexity and may influence postoperative outcomes [11,12,13].

Previous studies have primarily focused on surgical safety and short-term visual acuity outcomes in patients with congenital iris–choroidal coloboma [7,14,15]. Nevertheless, visual acuity alone is insufficient to fully characterize postoperative visual function. Despite uneventful surgery, postoperative optical quality may remain compromised in these patients, and the underlying mechanisms have not been fully elucidated. To date, comprehensive evaluations of surgical safety and efficacy are limited, particularly with respect to comparative analyses of postoperative IOL position and objective optical quality in eyes with iris–choroidal coloboma.

Therefore, this retrospective study aimed to evaluate short-term surgical outcomes, postoperative IOL alignment, and objective optical quality after phacoemulsification with IOL implantation in patients with congenital iris–choroidal coloboma and cataract. By analyzing postoperative IOL position, objective optical quality parameters, perioperative changes in best-corrected visual acuity (BCVA) and intraocular pressure (IOP), we sought to provide evidence to inform clinical management and surgical planning for this challenging group of patients.

## 2. Materials and Methods

### 2.1. Study Design and Participants

This retrospective comparative study was conducted at the Department of Cataract, Beijing Tongren Hospital, Capital Medical University, in adherence to the tenets of the Declaration of Helsinki. The study protocol was approved by the Ethics Committee of Beijing Tongren Hospital (Approval No. TREC2025-KY004).

Medical records of patients diagnosed with congenital iris–choroidal coloboma complicated by cataract who were evaluated for cataract surgery between January 2020 and October 2024 were retrospectively reviewed. Patients were included if they had a clinical diagnosis of congenital iris–choroidal coloboma confirmed by slit-lamp biomicroscopy and fundus examination, presented with visually significant cataract requiring surgical intervention, and underwent phacoemulsification with posterior chamber IOL implantation with complete data for the primary clinical and IOL-position outcomes. Eyes with a history of ocular trauma or previous intraocular surgery, corneal diseases affecting optical quality assessment, active intraocular inflammation, retinal detachment, or other retinal disorders unrelated to coloboma that could substantially influence postoperative visual outcomes were excluded. The age-related cataract group comprised consecutive eligible patients identified by chronological screening of the same surgeon’s cataract surgical records during the same study period; all met the same exclusion criteria, received the same IOL model, and underwent the same examinations, with selection independent of OPD-Scan III availability. To avoid inter-eye correlation, only one eye per patient was included in the analysis.

### 2.2. Preoperative Examination

Baseline demographic and clinical data were collected preoperatively, including sex, age at surgery, laterality (unilateral or bilateral involvement), and classification of iris and choroidal coloboma. All included eyes underwent comprehensive preoperative ophthalmologic examinations, including BCVA and IOP measurements, slit-lamp biomicroscopy, corneal endothelial cell density assessment, fundus photography, optical coherence tomography, and ocular ultrasonography. Axial length (AL) and anterior chamber depth (ACD) were measured using an optical biometer (IOLMaster 700, Carl Zeiss Meditec AG, Jena, Germany), and keratometric values (K1 and K2) were measured with a Scheimpflug tomographer (Pentacam HR, Oculus Optikgeräte GmbH, Wetzlar, Germany).

### 2.3. Surgical Procedure

All surgeries were performed under local anesthesia by the same experienced surgeon (X.S.) using a standardized phacoemulsification technique through a 2.4 mm corneal incision. A central continuous curvilinear capsulorhexis with a diameter of approximately 5.5 mm was performed. Given the limited zonular or capsular damage and preserved capsular support, no capsular tension rings (CTRs) or iris retractors were required in this cohort. Finally, a monofocal IOL (Hoya 250; HOYA Corporation, Tokyo, Japan) was implanted into the capsular bag.

### 2.4. Postoperative Examination

Postoperative assessments were performed at 1 month including BCVA, IOP, and objective evaluations of IOL position and optical quality. All patients were routinely followed for 6 months postoperatively, focusing on the assessment of complications and the need for further intervention.

Early postoperative IOL tilt and decentration were measured using anterior segment optical coherence tomography (AS-OCT, CASIA2, Tomey Corporation, Nagoya, Japan). All eyes were examined using the same automated three-dimensional AS-OCT protocol, with IOL tilt and decentration referenced to the corneal topographic axis. Image quality and automated segmentation were reviewed by two masked graders and corrected when necessary.

Objective optical quality parameters were assessed using a wavefront aberrometer (OPD-Scan III, NIDEK Co., Ltd., Gamagori, Japan), including modulation transfer function (MTF) and Strehl ratio (SR). Examinations were performed under standardized scotopic illumination with pharmacologic mydriasis and fixation on the internal target, and analyzed using a standardized 3.0 mm pupil diameter. One OPD-Scan III scan was obtained from each eye, and only measurements meeting the manufacturer-defined acquisition-quality criteria were included. Valid measurements were obtained in 17 coloboma eyes and 30 age-related cataract eyes. Specifically, MTF reflects the contrast transfer capability of the optical system across spatial frequencies, whereas SR, derived from the point spread function (PSF), quantifies the impact of optical aberrations on image quality. When combined, these parameters reflect the overall imaging quality of the optical system across all spatial frequencies and enable quantitative evaluation of objective optical performance [16]. Objective optical quality was analyzed only in eyes with valid OPD-Scan III measurements, and measurable and non-measurable coloboma eyes were compared for potential selection bias.

### 2.5. Coloboma Classification and Regression Analysis

According to the extent of iris and choroidal involvement, eyes in the coloboma group were further classified into four subtypes based on previously published classifications: isolated iris coloboma, iris coloboma involving the crystalline lens, iris–lens coloboma with choroidal involvement sparing the macula and optic disc, and iris–lens coloboma with choroidal involvement affecting the macula and/or optic disc [7]. Because the first two subtypes included few eyes, coloboma eyes were subsequently categorized according to the presence or absence of macular and/or optic-disc involvement.

Within the coloboma group, factors associated with postoperative BCVA were evaluated using multiple linear regression, including age at surgery, preoperative BCVA, and macular and/or optic-disc involvement. The associations of IOL tilt with macular and/or optic-disc involvement, AL, and ACD were evaluated in a single multivariable regression model, with all three predictors entered simultaneously.

### 2.6. Statistical Analysis

Statistical analyses were performed using SPSS software (version 26.0; IBM Corp., Armonk, NY, USA). Normality of continuous variables was assessed using the Shapiro–Wilk test. Parametric, nonparametric, or categorical tests were used as appropriate. BCVA and IOP were analyzed using age-adjusted mixed-effects models with group-by-time interactions and an unstructured residual covariance matrix, whereas MTF was analyzed using an age-adjusted mixed-effects model with a group-by-spatial-frequency interaction and an ARH (1) covariance structure. No additional random effects were specified. Model convergence and residual diagnostic plots showed no substantial violations of model assumptions. Multivariable regression analyses were performed for postoperative BCVA and IOL tilt, with model assumptions assessed. Effect estimates were reported with 95% confidence intervals where applicable. Between-group analyses of IOL tilt, decentration, MTF, and SR were adjusted for age, with additional adjustment for ACD in sensitivity analyses of IOL tilt and decentration. A two-sided *p* value < 0.05 was considered statistically significant.

## 3. Results

### 3.1. Clinical Characteristics

All 36 eligible coloboma eyes underwent attempted phacoemulsification and successfully completed posterior chamber IOL implantation; no planned procedures failed. During the same period, 31 patients (31 eyes) with age-related cataract undergoing the same surgical procedure were included as controls. As expected for the disease profile, patients in the congenital iris–choroidal coloboma group underwent cataract surgery at a younger age compared with those in the age-related cataract group (51.86 ± 12.92 vs. 65.87 ± 10.13 years, respectively; *p* < 0.001), whereas the sex distribution was comparable between the two groups (*p* = 0.892). The ACD was significantly shallower in the coloboma group than in the age-related cataract group (2.49 [2.18, 2.94] vs. 3.28 [2.97, 3.73] mm; *p* < 0.001). Within the coloboma group, defect types involving choroid were more prevalent than defects limited to the iris or lens zonular involvement. No significant differences were observed between the two groups in AL, K1, or K2 (all *p* > 0.05). Baseline demographic and clinical characteristics are summarized in Table 1.

### 3.2. Visual Outcomes and Intraocular Pressure

After adjustment for age, BCVA improved significantly at 1 month postoperatively compared with preoperative values in both groups (both *p* < 0.001). The group-by-time interaction was not significant (*p* = 0.419), indicating no significant difference in BCVA improvement between groups. However, adjusted BCVA remained significantly worse in the coloboma group at 1 month (between-group difference, 0.685 logMAR; 95% CI, 0.509–0.861; *p* < 0.001; Table 2).

No statistically significant change in IOP was detected between the preoperative and 1-month postoperative assessments in either group, with no significant difference in the magnitude of change between groups (*p* = 0.700). Nevertheless, IOP was higher in the coloboma group both preoperatively (adjusted difference, 1.871 mmHg; 95% CI, 0.456–3.286; *p* = 0.010) and postoperatively (adjusted difference, 2.128 mmHg; 95% CI, 0.885–3.371; *p* = 0.001) (Table 2).

### 3.3. Surgical and Postoperative Complications

Intraoperative complications occurred in 3 of 36 eyes (8.3%) in the coloboma group. Zonular dialysis occurred in all three eyes, two of which also developed anterior capsular tears. The capsular bag was stabilized with an ophthalmic viscosurgical device, and phacoemulsification was completed using reduced fluidic parameters, minimal lens rotation, and careful cortical aspiration to minimize further zonular stress and prevent extension of the capsular tears. Phacoemulsification and in-the-bag IOL implantation were successfully completed in all 36 eyes. As both the anterior capsular tears and inferior zonular dialysis were limited in extent and did not compromise capsular bag stability, no capsular tension ring was required.

All 36 eyes completed the 6-month postoperative follow-up. During this period, posterior capsule opacification developed in two eyes and was treated with Nd:YAG laser capsulotomy. No other postoperative complications were observed.

### 3.4. Postoperative IOL Tilt and Decentration

At 1 month postoperatively, eyes with congenital iris–choroidal coloboma showed greater IOL tilt and decentration than age-related cataract eyes. After adjustment for age, the between-group difference was 4.429° for IOL tilt (*p* < 0.001) and 0.183 mm for IOL decentration (*p* = 0.004). In an additional sensitivity analysis adjusting for both age and ACD, the between-group differences remained significant for IOL tilt (*p* < 0.001) and IOL decentration (*p* = 0.003) (Table 3). The three eyes with intraoperative complications were included in the primary analysis. After exclusion of these eyes, the differences remained significant for both IOL tilt (adjusted difference, 3.561°; 95% CI, 2.322–4.800; *p* < 0.001) and decentration (adjusted difference, 0.166 mm; 95% CI, 0.050–0.282; *p* = 0.006).

### 3.5. Postoperative Optical Quality

Due to poor fixation or nystagmus, valid OPD-Scan III measurements were available in 17 of 36 coloboma eyes and 30 of 31 age-related cataract eyes. Within the coloboma cohort, measurement failure was strongly associated with nystagmus (18/19 vs. 0/17; Fisher’s exact *p* < 0.001), whereas no significant differences were observed in the other assessed clinical characteristics between evaluable and non-evaluable eyes (all *p* > 0.05; Table S1). Accordingly, optical-quality analyses were limited to the evaluable subgroup.

**Figure 1:**
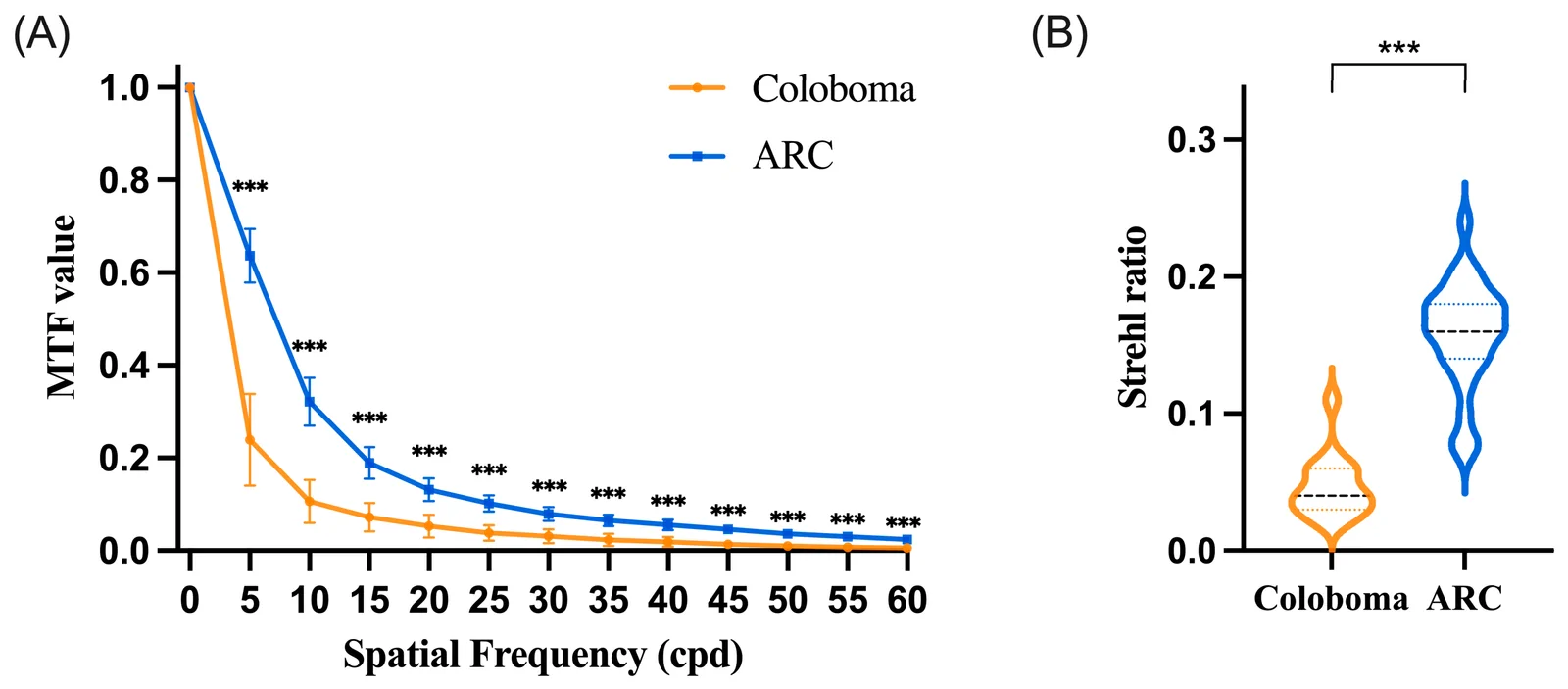
Postoperative objective optical quality in congenital iris–choroidal coloboma and age-related cataract eyes. (**A**) Modulation transfer function (MTF) curves in the coloboma group (*n* = 17) and age-related cataract (ARC) group (*n* = 30). Data are presented as mean with 95% confidence intervals. MTF values from 5 to 60 cycles/degree were analyzed using an age-adjusted linear mixed-effects model accounting for repeated measurements across spatial frequencies. A significant group-by-spatial-frequency interaction was observed (*p* < 0.001), and between-group differences were significant at all analyzed spatial frequencies after Bonferroni correction (*** *p* < 0.001). (**B**) Postoperative Strehl ratio (SR). Violin plots show the median and interquartile range. The SR was significantly lower in the coloboma group than in the ARC group after adjustment for age (*** *p* < 0.001).

MTF decreased with increasing spatial frequency in both groups. An age-adjusted mixed-effects model showed a significant group-by-spatial-frequency interaction (*p* < 0.001). MTF was significantly lower in the coloboma group at all analyzed spatial frequencies from 5 to 60 cycles/degree, with all between-group comparisons remaining significant after Bonferroni correction (all *p* < 0.001). The adjusted difference ranged from −0.399 (95% CI, −0.515 to −0.282) at 5 cycles/degree to −0.020 (95% CI, −0.030 to −0.010) at 60 cycles/degree (Figure 1A).

Similarly, SR values were lower in the coloboma group than in age-related cataract eyes (Figure 1B). This difference remained significant after adjustment for age, with an adjusted between-group difference of −0.106 (95% CI, −0.130 to −0.081; *p* < 0.001).

### 3.6. Factors Associated with Postoperative Outcomes

In multivariable linear regression analysis, macular and/or optic-disc involvement was significantly associated with worse postoperative BCVA (*B* = 0.832; 95% CI, 0.556–1.108; *p* < 0.001), whereas age at surgery and preoperative BCVA were not significantly associated with postoperative BCVA. The model explained 63.8% of the variance in postoperative BCVA (*R*^2^ = 0.638; adjusted *R*^2^ = 0.604).

For postoperative IOL tilt, multivariable analysis with robust covariance estimation showed that macular and/or optic-disc involvement was associated with greater tilt (*B* = 4.750°; 95% CI, 0.535–8.964; *p* = 0.027). No significant associations were observed for AL or ACD (Table S2).

## 4. Discussion

In this retrospective comparative study, we systematically evaluated short-term visual outcomes, postoperative IOL alignment, and objective optical quality after phacoemulsification with IOL implantation in eyes with congenital iris–choroidal coloboma complicated by cataract. Our findings showed that while surgery successfully improved visual acuity with no statistically significant change in IOP, colobomatous eyes exhibited greater IOL tilt and decentration and poorer objective optical quality in the evaluable subgroup compared with age-related cataracts at 1 month postoperatively. Furthermore, multivariable analysis revealed that involvement of the macula and/or optic disc was associated with worse postoperative BCVA and greater postoperative IOL tilt.

At present, surgery remains the only effective treatment for iris–choroidal coloboma complicated by cataract [7]. Common procedures include phacoemulsification, small-incision cataract surgery and conventional extracapsular cataract extraction [12], among which phacoemulsification is the most widely used and effective technique for improving BCVA [8]. For example, Phylactou et al. performed phacoemulsification combined with IOL implantation in 41 eyes with iris–choroidal coloboma. The preoperative best-corrected distance visual acuity was 0.90 ± 0.55 logMAR, which improved to 0.62 ± 0.49 logMAR at the final follow-up (*p* < 0.001) [7]. Similarly, Wang et al. followed 20 eyes after phacoemulsification combined with IOL implantation and reported that the median preoperative best-corrected distance visual acuity was 1.70 (1.08, 2.10) logMAR, which improved to 0.90 (0.50, 1.00) logMAR postoperatively (*p* < 0.01) [14]. Similarly, BCVA improved significantly after surgery in coloboma eyes in our study (*p* < 0.001). However, both preoperative and postoperative BCVA remained worse than those in age-related cataract eyes after adjustment for age. This finding is biologically plausible, given the congenital abnormalities of the retina, retinal pigment epithelium, choroid, and optic disc, including thinning of the neurosensory retina, absence of the choriocapillaris, and morphological abnormalities of the retinal pigment epithelium, which lead to reduced retinal image quality [17]. These changes are more pronounced when the defect involves the macula and optic disc [8,18]. Moreover, structural abnormalities of the posterior segment may cause irreversible damage to photoreceptor function and interfere with visual signal transmission, thereby limiting postoperative visual recovery [1].

However, despite these encouraging visual acuity outcomes, few studies have evaluated postoperative objective optical quality in patients with iris–choroidal coloboma. In the present study, postoperative optical quality was assessed at 1 month using OPD-Scan III, including objective analysis of MTF and SR [19]. In the evaluable subgroup, MTF decreased with increasing spatial frequency, indicating superior contrast transmission at lower spatial frequencies, in line with the frequency-dependent pattern described by Zhu et al. [20]. Furthermore, coloboma eyes showed consistently lower MTF values from 5 to 60 cycles/degree and lower SR after adjustment for age (all *p* < 0.001). These findings indicate that although BCVA improved significantly after surgery, postoperative optical quality in the evaluable coloboma subgroup remains relatively poor. Chen et al. reported that axial length is an important factor associated with postoperative IOL tilt after phacoemulsification with IOL implantation [21]. This malposition may impair the optical performance of the IOL and thereby contribute to poorer postoperative optical quality [19]. In addition, other studies have reported that pupil size and IOL decentration may also influence postoperative visual performance [22,23,24]. In eyes with congenital choroidal coloboma, when the defect involves the pupillary region, impaired pupillary dilation may reduce retinal illumination, thereby further compromising optical quality. In addition to posterior segment abnormalities, stretching of lax or defective zonular fibers during surgery may result in greater postoperative IOL tilt and decentration, which further impairs optical performance.

Although some researchers have classified iris and choroidal coloboma based on defect extent, it remains unclear whether the severity of choroidal defects is correlated with postoperative visual outcomes [4,25]. In this context, our study demonstrated that age and preoperative BCVA were not independently associated with postoperative BCVA, whereas macular and/or optic-disc involvement was significantly associated with worse postoperative BCVA. This finding may be related to congenital developmental abnormalities and frequent coexistence of amblyopia. Consequently, when the defect involves the optic disc and macula, visual improvement after cataract surgery may be limited compared with eyes without posterior pole involvement. From a practical standpoint, evaluating posterior pole involvement is therefore important for surgeons’ preoperative decision-making and prognostic counseling, as it was associated not only with worse postoperative BCVA but also with greater postoperative IOL tilt.

Surgical safety is another critical consideration in this patient population. Previous studies have shown that phacoemulsification can maintain relatively stable IOP both intraoperatively and postoperatively [14]. For instance, Thomas et al. analyzed IOP changes during different surgical stages and provided evidence supporting the intraoperative safety of this procedure [26]. Through dynamic balance between irrigation and aspiration, IOP fluctuations can be effectively reduced, which may help support intraoperative safety. Wang et al. reported stable preoperative and postoperative IOP after phacoemulsification with IOL implantation in eyes with iris–choroidal coloboma [14]. Similarly, no statistically significant change in IOP was detected in either group in our study, although coloboma eyes exhibited consistently higher IOP values than age-related cataract eyes after adjustment for age (*p* < 0.05). However, the present data do not permit determination of the underlying anatomical or physiological explanation.

To date, few studies have evaluated postoperative IOL position, particularly tilt and decentration, in patients with iris–choroidal coloboma. In the present study, AS-OCT was performed at 1 month after surgery and showed that the median postoperative IOL tilt and decentration in the coloboma group were 7.60° (5.4, 10.3°) and 0.40 mm (0.21, 0.62 mm), respectively. After adjustment for age, both IOL tilt and decentration remained significantly greater in the coloboma group than in the age-related cataract group (*p* < 0.001 and *p* = 0.004, respectively). These findings suggest that congenital iris–choroidal coloboma is associated with less optimal early postoperative IOL alignment, which may reflect underlying zonular abnormalities [7]. This association between posterior pole involvement and greater IOL tilt may reflect more extensive congenital ocular abnormalities in eyes with posterior pole involvement, including possible zonular or capsular abnormalities. Our findings in age-related cataract eyes are generally consistent with previous reports. Timo Eppig et al. reviewed studies published over a ten-year period and reported a mean IOL tilt of 2.62 ± 1.14° (range, 0.20–8.17°) and a mean decentration of 0.30 ± 0.16 mm (range, 0.00–1.09 mm) after cataract surgery [27]. Excessive IOL tilt or decentration may compromise optical quality [21,28], particularly when tilt exceeds 7° or decentration exceeds 0.4 mm [29].

Therefore, from a surgical perspective, particular attention may be warranted in patients with congenital iris–choroidal coloboma complicated by cataract, especially in eyes with macular and/or optic-disc involvement. Continuous curvilinear capsulorhexis should be carefully performed with an appropriate size and intact contour, as an excessively large capsulorhexis may increase the risk of anterior capsular tearing, whereas an overly small capsulorhexis may hinder IOL implantation and increase the risk of postoperative IOL displacement [21]. During surgery, the surgeon should thoroughly remove cortical material and perform gentle, controlled maneuvers under viscoelastic protection to minimize further zonular damage. When necessary, adjunctive devices such as iris hooks or capsular tension rings may be used to improve capsular bag stability and promote better postoperative IOL centration [12,15].

Previous studies have described the ocular characteristics and the intraoperative complications during phacoemulsification in coloboma eyes, which can be technically challenging due to poor zonular support, inadequate pupillary dilation, and microphthalmia. Sahay et al. and Phylactou et al. reported complications during cataract surgery in eyes with iris–choroidal coloboma, including posterior capsule rupture, anterior capsule tears, and zonular dialysis [7,30]. Similar complications were observed in our cohort, with zonular dialysis occurring in three eyes, two of which also developed anterior capsular tears, as detailed in the Results. These findings highlight the importance of careful perioperative assessment of capsular bag integrity, zonular status, and the extent of choroidal coloboma, as well as meticulous intraoperative manipulation, to reduce the risk of intraoperative complications.

To our knowledge, this study provides a comprehensive evaluation of short-term surgical outcomes, postoperative IOL alignment, and objective optical quality in patients with congenital iris–choroidal coloboma complicated by cataract, with objective optical quality additionally assessed in the evaluable subgroup. By comparatively analyzing postoperative IOL position and, in the evaluable subgroup, objective optical quality, our findings help address an important gap in the surgical evaluation of these eyes and may provide useful guidance for clinical practice. Preoperative evaluation of zonular status and posterior pole involvement may help optimize surgical planning and prognostic counseling.

However, this study was retrospective, and preoperative optical quality was not systematically assessed, limiting evaluation of preoperative to postoperative changes in optical quality. In addition, underrepresentation of eyes with nystagmus in the evaluable subgroup may have introduced selection bias and limited the generalizability of the optical-quality findings. Furthermore, age and ACD may influence postoperative outcomes and should be considered. Residual confounding cannot be completely excluded despite statistical adjustment. In addition, the relatively small sample size may reduce statistical power and limit generalizability. Although clinical follow-up for postoperative complications extended to 6 months, postoperative follow-up for AS-OCT and OPD-Scan III examinations was limited to 1 month. Therefore, the present findings describe early postoperative IOL alignment and short-term optical outcomes rather than longitudinal IOL stability or longer-term functional outcomes. Long-term functional outcomes require further investigation in larger prospective studies with extended follow-up.

## 5. Conclusions

In conclusion, phacoemulsification combined with IOL implantation was associated with short-term visual acuity improvement in eyes with congenital iris–choroidal coloboma. However, these eyes exhibited greater early postoperative IOL tilt and decentration than age-related cataract eyes, while the evaluable subgroup showed poorer objective optical quality. Larger prospective studies with extended follow-up are needed to further validate these findings.

## Figures and Tables

**Table 1:** Baseline demographic and clinical characteristics.

Variable	Coloboma Group (*n* = 36)	Age-Related Cataract Group (*n* = 31)	*p* Value
**Age at surgery (years)**	51.86 ± 12.92	65.87 ± 10.13	<0.001 ***
**Sex, n (%)**			0.892
Male	11 (30.56%)	9 (29.03%)	
Female	25 (69.44%)	22 (70.97%)	
**Extent of coloboma, n (%)**			-
Isolated iris coloboma	1 (2.78%)	-	
Iris–lens coloboma	4 (11.11%)	-	
Iris–lens coloboma with choroidal involvement (without macular/optic disc involvement)	17 (47.22%)	-	
Iris–lens coloboma with choroidal involvement (with macular/optic disc involvement)	14 (38.89%)	-	
**AL (mm)**	24.04 (22.58, 25.69)	24.20 (23.53, 26.60)	0.157
**ACD (mm)**	2.49 (2.18, 2.94)	3.28 (2.97, 3.73)	<0.001 ***
**K1 (D)**	43.47 ± 2.01	43.58 ± 1.69	0.802
**K2 (D)**	45.09 ± 1.88	44.86 ± 1.59	0.584

Note: Data are presented as mean ± standard deviation (SD) for normally distributed variables (age at surgery, K1, and K2) and as median (P25, P75) for non-normally distributed variables (AL and ACD). Comparisons between groups were performed using the independent-samples *t* test for normally distributed variables, the Mann–Whitney *U* test for non-normally distributed variables, and the chi-square test or Fisher’s exact test for categorical variables, as appropriate. *** *p* < 0.001. Abbreviations: AL, axial length; ACD, anterior chamber depth; K1, flat keratometry; K2, steep keratometry; P25, 25th percentile; P75, 75th percentile.

**Table 2:** Age-adjusted changes in BCVA and IOP from the preoperative to the 1-month postoperative assessment.

Outcome	Group	Eyes (*n*)	Preoperative Adjusted Mean (95% CI)	Postoperative Adjusted Mean (95% CI)	Within-Group Change (95% CI)	Within-Group *p* Value	Between-Group Difference in Change (95% CI)	Group × Time *p* Value
BCVA, logMAR	Coloboma	36	1.187 (0.980–1.395)	0.702 (0.528–0.877)	0.485 (0.279–0.691)	<0.001	0.085 (−0.126–0.296)	0.419
	Age-related cataract	31	0.417 (0.374–0.460)	0.017 (−0.001–0.035)	0.400 (0.350–0.450)	<0.001		
IOP, mmHg	Coloboma	36	16.406 (15.371–17.441)	16.398 (15.457–17.340)	0.008 (−1.135–1.150)	0.989	−0.257 (−1.585–1.072)	0.700
	Age-related cataract	31	14.535 (13.682–15.388)	14.270 (13.570–14.971)	0.265 (−0.451–0.980)	0.457		

Note: Data are presented as age-adjusted estimated marginal means with 95% confidence intervals (CIs). Changes in BCVA and IOP were analyzed using mixed-effects models including group, time, the group-by-time interaction, and age. Within-group change was calculated as the preoperative value minus the postoperative value. The group-by-time interaction tests whether the magnitude of change differed between groups. Abbreviations: BCVA, best-corrected visual acuity; IOP, intraocular pressure; CI, confidence interval.

**Table 3:** IOL Tilt and Decentration at 1 Month After Surgery.

Parameter	Coloboma Group (*n* = 36)	Age-Related Cataract Group (*n* = 31)	Age-Adjusted Difference (95% CI), *p* Value	Age- and ACD-Adjusted Difference (95% CI), *p* Value
IOL tilt (°)	7.60 (5.4, 10.3)	4.10 (3.4, 5.0)	4.429 (2.721–6.136), *p* < 0.001 ***	4.971 (3.350–6.593), *p* < 0.001 ***
IOL decentration (mm)	0.40 (0.21, 0.62)	0.14 (0.10, 0.35)	0.183 (0.062–0.305), *p* = 0.004	0.237 (0.083–0.391), *p* = 0.003

Note: Data are presented as median (IQR). Adjusted differences represent the coloboma group minus the age-related cataract group. The primary analyses were adjusted for age, and sensitivity analyses were additionally adjusted for ACD. IOL tilt was analyzed using a generalized linear model with a normal distribution, identity link, and robust covariance estimator; IOL decentration was analyzed using ANCOVA. *** *p* < 0.001. Abbreviations: IQR, interquartile range; ACD, anterior chamber depth; IOL, intraocular lens; ANCOVA, analysis of covariance; CI, confidence interval.

## Data Availability

The data supporting the findings of this study are available from the corresponding author upon reasonable request.

## Supplementary Materials

The following supporting information can be downloaded at: https://www.mdpi.com/article/10.3390/jcm15176616/s1, Table S1. Comparison of clinical characteristics between coloboma eyes with and without valid OPD-Scan III measurements. Table S2. Multivariable analyses of factors associated with postoperative BCVA and IOL tilt in the coloboma group.
