# Supplementary material for: Cataract Surgery in Eyes with Congenital Iris–Choroidal Coloboma: Quantitative Assessment of Intraocular Lens Alignment and Optical Quality
Source: J Clin Med. 2026 Aug 27;15(17):6616. doi: 10.3390/jcm15176616 (PMC13566231; doi:10.3390/jcm15176616)
Supplement: Supplementary file 1 [file jcm-15-06616-s001.zip › jcm-4446702-supplementary.pdf]

Journal of Clinical Medicine

MDPI

Table S1.Comparison of clinical characteristics between coloboma eyes with and without valid OPD-Scan III measurements.

Variable	With Valid OPD-Scan III measurements (n = 17)	Without Valid OPD-Scan III measurements (n = 19)	P value
**Preoperative characteristics**			
Age at surgery (years)	52.47 ± 10.71	51.32 ± 14.89	0.793
Sex (n (%))			0.721
male	6 (35.3%)	5 (26.3%)	
female	11 (64.7%)	14 (73.7%)	
Preoperative BCVA (logMAR)	1.00 (0.75,1.30)	1.30 (0.52,1.70)	0.452
Preoperative IOP (mmHg)	17.15 ± 2.93	15.57 ± 2.83	0.109
AL (mm)	24.04 (22.56,26.02)	24.04 (22.51,25.71)	0.975
ACD (mm)	2.49 (2.09,3.06)	2.49 (2.24,2.90)	0.707
K1 (D)	43.07 ± 1.82	43.83 ± 2.16	0.263
K2 (D)	44.68 ± 1.77	45.45 ± 1.94	0.225
Macular and/or optic disc involvement (n (%))	6 (35.3%)	8 (42.1%)	0.742
Nystagmus (n (%))	0 (0.0%)	18 (94.7%)	<0.001***
**Postoperative characteristics**			
Postoperative BCVA at 1 month (logMAR)	0.70 (0.19,1.30)	0.52 (0.22,1.30)	0.778
Postoperative IOP at 1 month (mmHg)	16.67 ± 2.83	15.99 ± 2.64	0.457
IOL tilt at 1 month (°)	7.10 (5.45,8.65)	7.60 (5.20,10.30)	0.415
IOL decentration at 1 month (mm)	0.42 ± 0.26	0.47 ± 0.21	0.509

Note: Data are presented as mean ± SD, median (IQR), or n (%), as appropriate. Continuous variables were compared using the independent-samples t test or Mann–Whitney U test according to data distribution. Categorical variables were compared using Fisher's exact test.*** $P < 0.001$.

Table S2. Multivariable analyses of factors associated with postoperative BCVA and IOL tilt in the coloboma group.

Outcome	Predictor	B	95% CI	*P* value
**Postoperative BCVA**	Age at surgery, years	−0.004	−0.013 to 0.006	0.422
	Preoperative BCVA, logMAR	0.067	−0.143 to 0.276	0.520
	Macular and/or optic disc involvement	0.832	0.556 to 1.108	<0.001
**Postoperative IOL tilt**				
	Macular and/or optic disc involvement	4.750	0.535 to 8.964	0.027
	Axial length, mm	−0.795	−1.681 to 0.091	0.079
	Anterior chamber depth, mm	0.716	−1.811 to 3.242	0.579

Note: B represents the unstandardized regression coefficient. All analyses included 36 coloboma eyes. Macular and/or optic-disc involvement was coded as 1 = affected and 0 = unaffected, with unaffected eyes as the reference group. Postoperative BCVA was analyzed using multiple linear regression. Postoperative IOL tilt was analyzed using a generalized linear model with a normal distribution, identity link, and robust covariance estimator because the residual normality assumption of the corresponding ordinary least-squares model was not satisfied.*** $P < 0.001$.
